# Papuloerythroderma of Ofuji associated with sternoclavicular arthritis and successful treatment with cyclosporine

**DOI:** 10.1016/j.jdcr.2022.07.004

**Published:** 2022-07-09

**Authors:** Eri Kasai, Koji Habe, Yoshiaki Matsushima, Makoto Kondo, Keiichi Yamanaka

**Affiliations:** Department of Dermatology, Mie University, Graduate School of Medicine, Tsu, Japan

**Keywords:** papuloerythroderma, sternoclavicular arthritis, cyclosporine, PPP, palmoplantar pustulosis, SAPHO, synovitis, acne, pustulosis, hyperostosis, osteitis

## Introduction

Papuloerythroderma of Ofuji is characterized by polygonal, flat-topped, erythematous-brown papules that coalesce into sheets that often cover the entire skin surface but without skin rash in large intertriginous areas such as the abdomen, antecubital fossa, popliteal fossa, and inguinal area, called as the deck-chair sign. Papuloerythroderma may be drug-induced or primary; however, it can be a paraneoplastic finding in the setting of malignancy. The range of oncotic disorders found in association with papuloerythroderma includes gastric carcinomas, hepatocellular carcinoma, adenocarcinoma, and cutaneous T-cell lymphoma.^1^

Sternoclavicular arthritis is evaluated using radiography, computed tomography, magnetic resonance imaging, and bone scintigraphy. Early diagnosis and treatment are critical because sternoclavicular arthritis may progress to irreversible bone and joint damage. Rheumatoid arthritis, spondyloarthritis (SpA), SAPHO (synovitis, acne, pustulosis, hyperostosis, osteitis) syndrome, and palmoplantar pustulosis (PPP) are well-known diseases that cause sternoclavicular arthritis. In SAPHO syndrome, the “bull's head” pattern of high tracer uptake in the sternoclavicular joint and angulus sterni on bone scintigraphy are specific.^2^^,^^3^

## Case presentation

**Fig 1:**
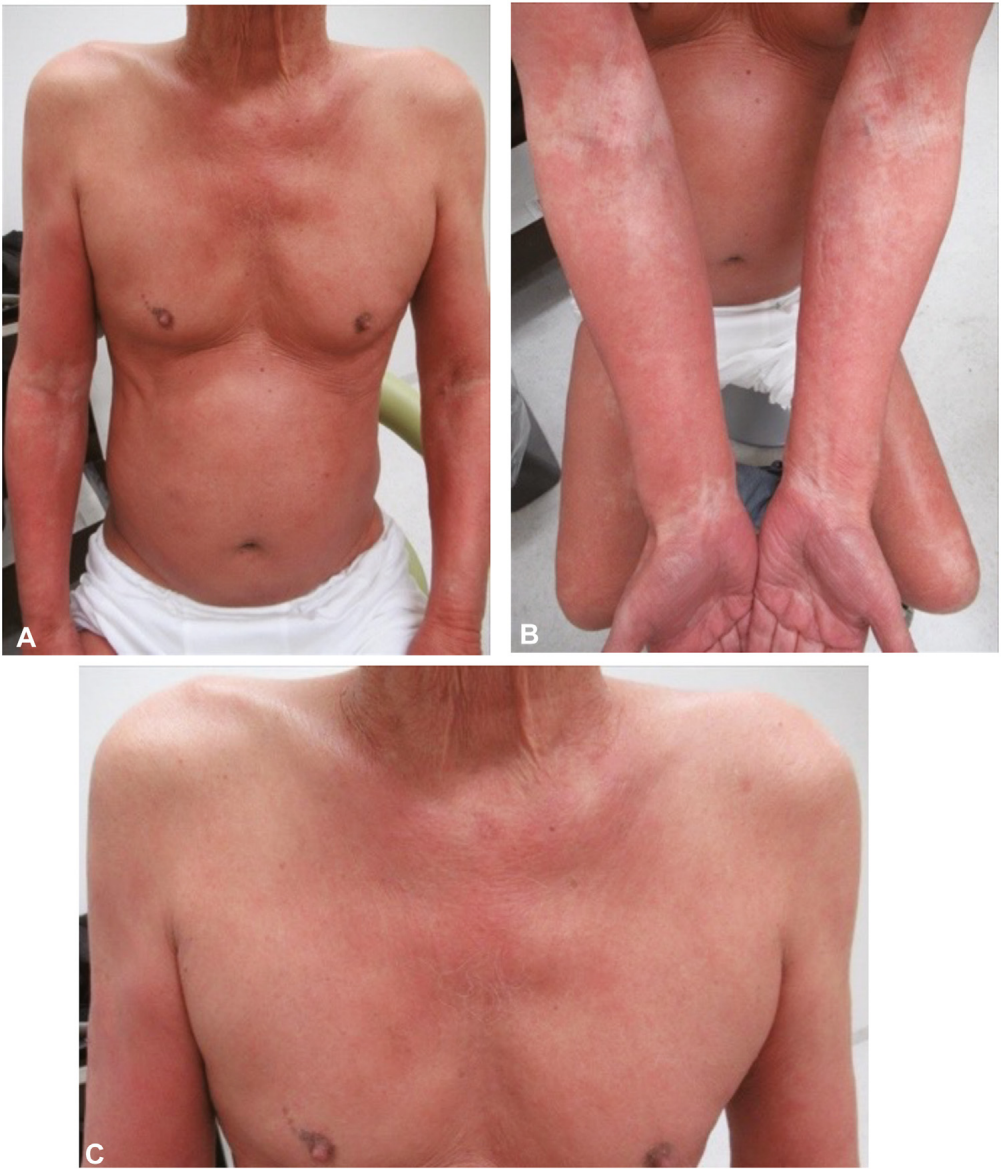
The clinical picture of a 79-year-old male patient at the first consultation. **A,** He had generalized 2 mm erythematous macules that coalesced on the trunk and extremities sparing the face. **B,** The antecubital fossa and wrist flexures were spared (deck-chair sign). **C,** The swelling and redness from the sternoclavicular joint to the sternum were noticed.

A 79-year-old man with a history of atherosclerotic disease was referred to our department for the treatment of rash. The patient was taking benidipine (dihydropyridine calcium channel blocker), atorvastatin calcium, lansoprazole, diltiazem, magnesium oxide, azilsartan (angiotensin II receptor antagonist), and aspirin. Six months prior to our consultation, erythema appeared throughout his body, and treatment with topical steroids and changes in oral medications for the possibility of drug eruption led to no improvement. Swelling and pain at the sternoclavicular joint also appeared. He had generalized 2 mm erythematous macules that coalesced on the trunk and extremities sparing the face (Fig 1). The antecubital fossa and wrist flexures were spared (deck-chair sign). Swelling of the median sternum and bilateral sternoclavicular joints was also noted.

**Fig 2:**
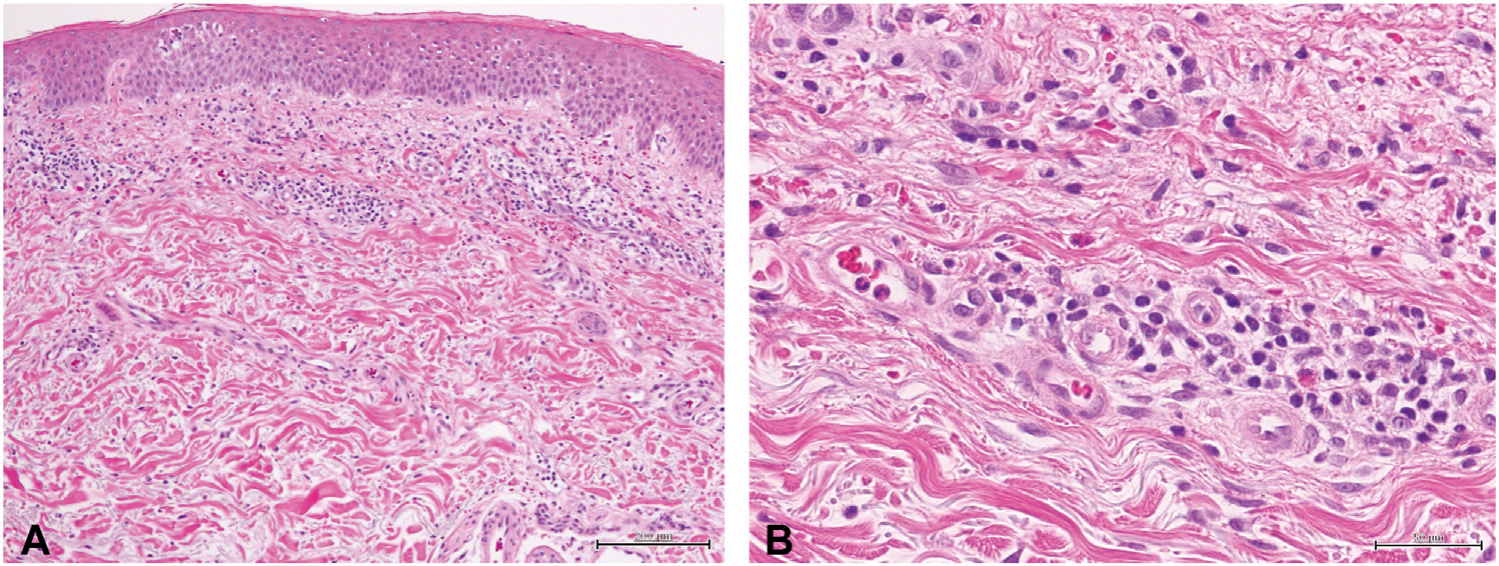
A skin biopsy from right thigh. **A,** Skin biopsy revealed epidermal spongiosis (hematoxylin and eosin staining [HE], bar 200 μM). **B,** There is a superficial perivascular lymphocytic infiltrate with a few eosinophils (HE, bar 50 μM).

**Fig 3:**
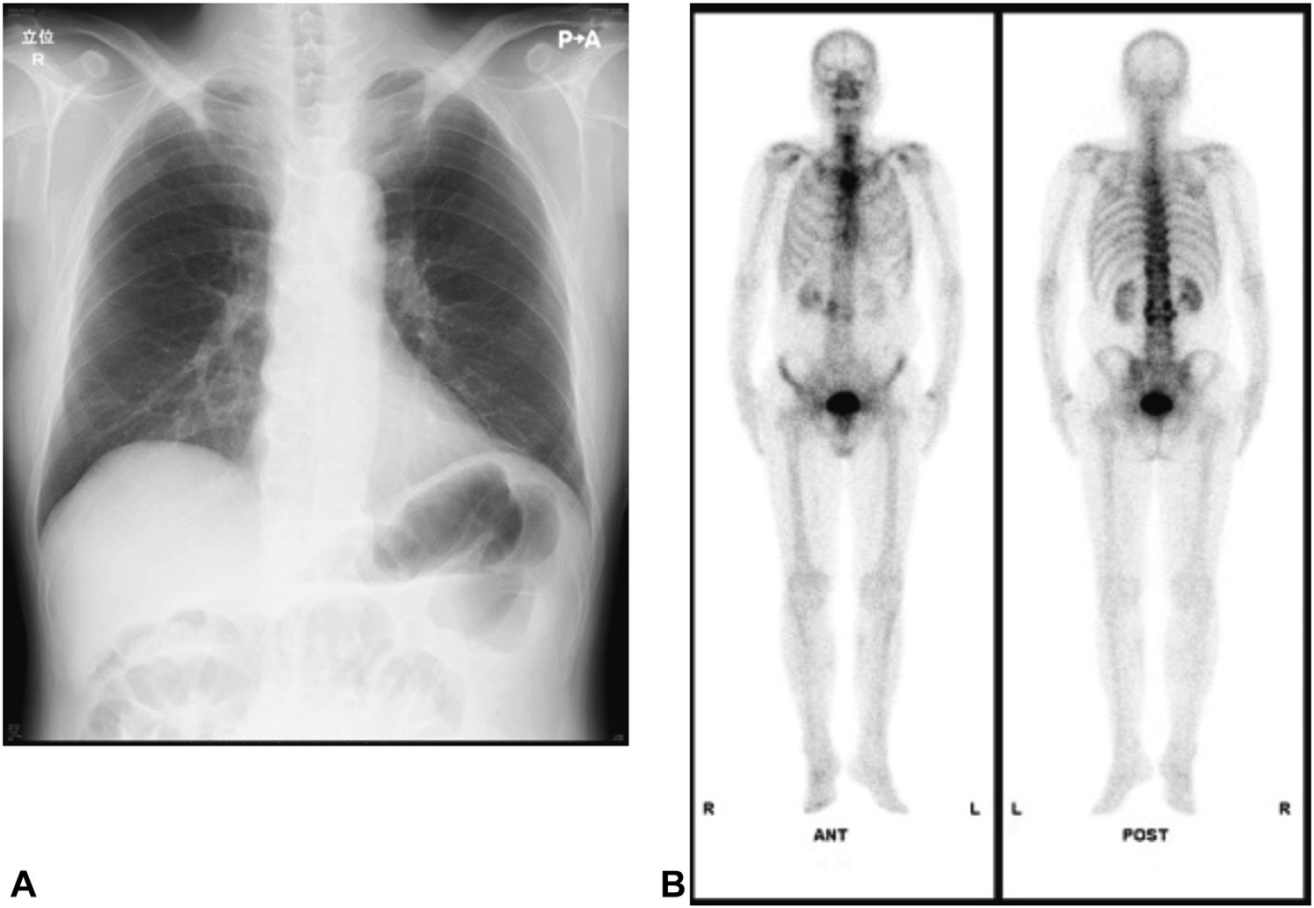
Chest X-ray showed bony hyperplasia of the sternoclavicular joint (**A**). Bone scintigraphy showed osteosclerosis changes were seen in the bilateral sternoclavicular joints, sternal pattern, and the first and second sternal rib joints and sclerotic changes in the anterior surfaces of the cervical to lumbar vertebral bodies (**B**).

Laboratory tests revealed eosinophilia and elevation of serum thymus and activation-regulated chemokine (TARC) level: white blood cell count 10,740/μL (normal, 3300-8600/μL), neutrophils 6497/μL (1900-6700/μL), eosinophils 1020/μL (70-450/μL), TARC 17,350pg/ml (<450pg/mL), total IgE 69 mg/dL (0-170 mg/dL), and C-reactive protein 0.11 mg/dL (<0.03 mg/dL). Skin biopsy revealed epidermal spongiosis and a superficial perivascular lymphocytic infiltrate with a few eosinophils (Fig 2, *A* and *B*). Chest radiography revealed bony hyperplasia of the sternoclavicular joint (Fig 3, *A*). Bone scintigraphy showed osteosclerotic changes in the bilateral sternoclavicular joints, sternal pattern, and the first and second sternal rib joints and sclerotic changes in the anterior surfaces of the cervical to lumbar vertebral bodies. No abnormal accumulation was observed in the sacroiliac joint (Fig 3, *B*).

His presentation including erythroderma, deck-chair sign, and elevated eosinophil count was most consistent with a diagnosis of papuloerythroderma. Screening for malignancy by computed tomography of the whole body and upper and lower endoscopy were negative. There was no lumbar stiffness or pain and no sacroiliac arthritis. Cutaneous symptoms such as acne or PPP were not observed during the course of the disease.

**Fig 4:**
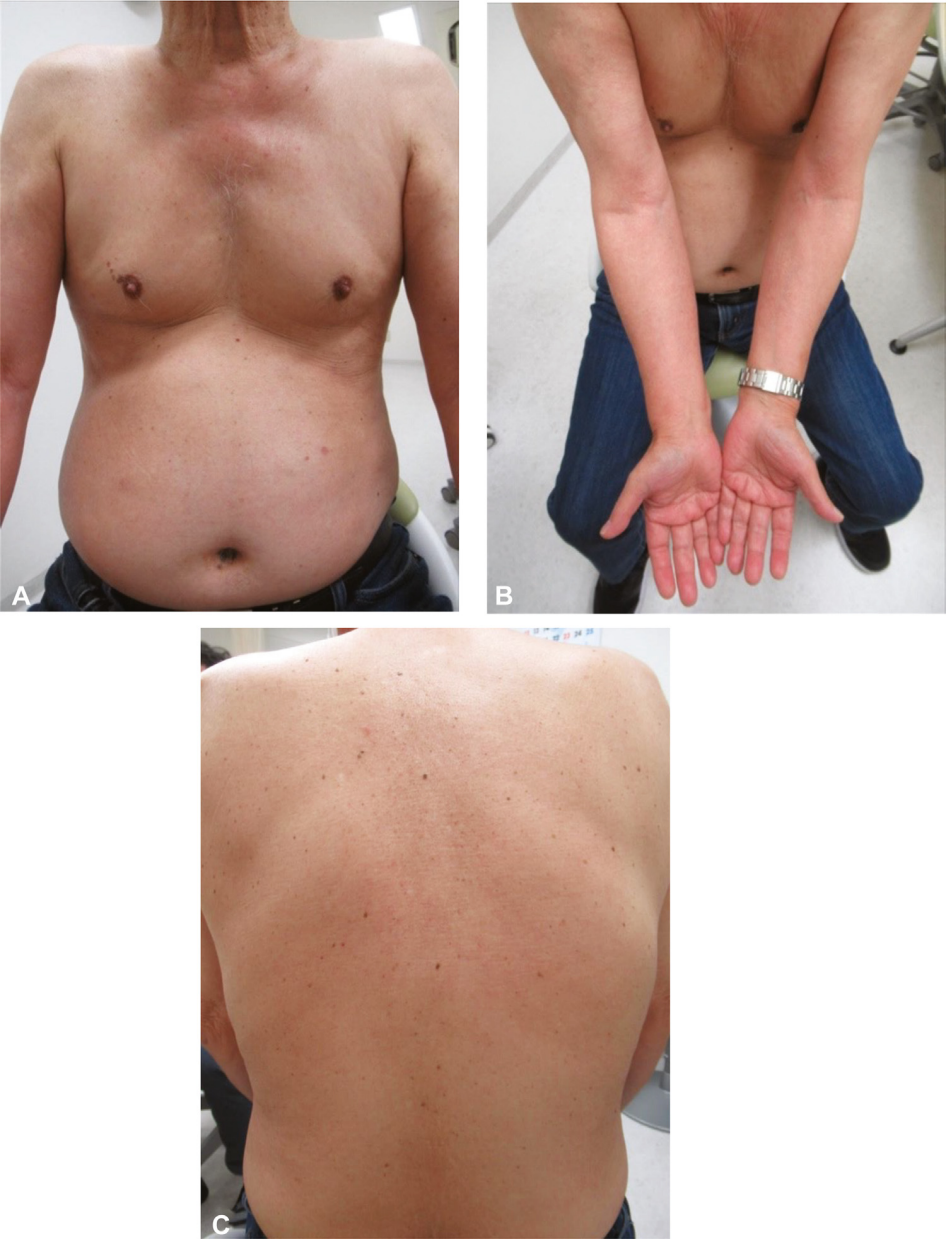
No recurrence of skin rash after resuming the treatment with cyclosporine. **A,** chest and abdomen; **B,** cubital fossa; **C,** back.

Th2-related cytokine, TARC, increases in various atopic diseases and correlates with disease severity. Based on the elevated serum TARC levels, the patient was considered to have Th2 cytokine-dominant skin lesions, and treatment was initiated with 125 mg/day (2 mg/kg) of cyclosporine. After 3 months of treatment, the erythema and papules resolved, and the joint symptoms improved. Laboratory tests showed marked improvement with an eosinophil count of 220/μL and TARC level of 407pg/ml; therefore, the dose of cyclosporine was reduced to 100 mg/day. Cyclosporine was discontinued after 1 month. However, 1 month after discontinuation of cyclosporine, the patient showed relapse of pruritus and full skin rash and elevated eosinophil count of 1734/μL and serum TARC level of 10,120pg/ml. The patient resumed treatment with 125 mg/day of cyclosporine. One month after resumption of treatment, the skin rash improved. Two months after resumption, the eosinophil count and serum TARC level improved to 182/μL and 237pg/ml, respectively, and chest radiography revealed improved sternal ossification. Thereafter, the patient was treated with maintenance cyclosporine and no relapse was observed (Fig 4).

## Discussion

The patient presented with an erythrodermic state of full skin rash with pruritus, deck-chair sign, and elevated eosinophil count, which led to the diagnosis of papuloerythroderma. There were no malignant complications or potential causative agents, and idiopathic papuloerythroderma was suspected. Periodic surveillance for malignancy is necessary because malignancy may occur after skin findings. Additionally, our case was complicated by sternoclavicular arthritis. Rheumatoid arthritis, spondyloarthritis, SAPHO syndrome, and PPP are diseases that cause sternoclavicular arthritis. In our case, laboratory tests and other clinical findings were negative for any of the aforementioned diseases. Bone scintigraphy showed findings similar to the “bull's head” pattern. It has been reported that 5% of patients with SAPHO syndrome have no skin lesions and 5.8% have preceding joint symptoms.^4^ Therefore, SAPHO syndrome cannot be completely ruled out. The possibility of PPP also cannot be completely excluded, as there are also reported cases of PPP preceded by joint symptoms. However, there have been no reports of any complications of papuloerythroderma with rheumatoid arthritis, spondyloarthritis, SAPHO syndrome, or PPP.

Treatment of papuloerythroderma, if secondary, includes treatment of the underlying malignancy and removal of the drug that induces skin rash. Patients with idiopathic disease currently have no gold standard therapy; however, previous cases have received treatment with photochemotherapy with psoralen and UVA, oral steroid courses, cyclosporine, and dupilumab.^5^ A 9-month course of cyclosporine at 3 mg/kg led to the complete resolution of skin lesions with some residual post-inflammatory changes.^6^ In our case, cyclosporine was effective against both skin lesions and arthritis. Skin lesions and arthritis were consistent with the pre- and post-treatment course of the disease, and the patient responded well to treatment. This clinical course strongly suggests that arthritis and bone lesions are associated with papuloerythroderma.

Here, we report a case of papuloerythroderma complicated by sternoclavicular arthritis. Since the symptoms of arthritis appeared after the skin rash and both the skin lesions and arthritis improved simultaneously, we consider that the patient had arthritis and bone lesions related to papuloerythroderma. There are no reports of papuloerythroderma complicated by bone lesions, and future case series are needed.

## Conflicts of interest

None disclosed.
